# Placental Histopathologic Lesions Associated With Stillbirth: A Systematic Review

**DOI:** 10.7759/cureus.106826

**Published:** 2026-04-10

**Authors:** Shrein Farouk Okasha Elsayed, Safaa Abdou El Sheshtawy, Lamiaa Hassan Hassan, Ayat Abdalwahid Gasmalseed Ahmed, Shaymaa Mohamed Assal, Nidal Hassan Sidahmed, Asseem Gamaz

**Affiliations:** 1 Obstetrics and Gynaecology, Corniche Hospital, Abu Dhabi, ARE; 2 Obstetrics and Gynaecology, Mediclinic Airport Road Hospital, Abu Dhabi, ARE; 3 Obstetrics and Gynaecology, Faculty of Medicine and Health Sciences, Omdurman Islamic University, Omdurman, SDN; 4 Obstetrics, Emirates Hospitals Day Surgery, Abu Dhabi, ARE; 5 Obstetrics and Gynaecology, Alshaya Medical Complex, Ar Rass, SAU

**Keywords:** inflammation, placental pathology, stillbirth, vascular malperfusion, villous maturation

## Abstract

Stillbirth is a major contributor to perinatal mortality. Placental examination is central to stillbirth evaluation. Published evidence is heterogeneous in lesion taxonomy, sampling approaches, comparator selection, and confounder control. We conducted a systematic review following PRISMA guidelines. We searched PubMed, Embase, and Scopus and screened reference lists to identify comparative observational studies. These studies evaluated placental histopathology in stillbirth compared with live birth or other clearly defined nonstillbirth comparator groups. We included studies that estimated stillbirth associations and those providing comparative insights into lesion reporting. Data were extracted on study design, stillbirth definition, comparator characteristics, placental sampling and reporting methods, lesion definitions, and reported association measures. Risk of bias was assessed using the Newcastle-Ottawa Scale. Due to substantial clinical and methodological heterogeneity, findings were synthesized narratively with structured tabulation. Across included studies, stillbirth was consistently associated with multidomain placental pathology. This was most prominent for vascular malperfusion and obstructive or hemorrhagic injury. Recurrent signals were also observed for villous maturation and hypoxia pattern abnormalities. Inflammatory lesions frequently co-occurred with vascular findings. These results support placental vascular pathology, villous developmental abnormalities, and inflammation as recurring domains linked to stillbirth. The findings underscore the need for more standardized definitions, sampling, and reporting. Standardization is needed to improve comparability and clinical interpretability.

## Introduction and background

Stillbirth, typically defined as fetal death after mid-gestation, with thresholds varying by jurisdiction, remains a major and often preventable contributor to perinatal mortality worldwide. It reflects complex interactions among maternal conditions, fetal vulnerability, and placental function [1-4]. Global initiatives emphasize that reducing stillbirths requires not only improvements in antenatal and intrapartum care but also stronger diagnostic attribution to identify modifiable pathways and inform recurrence-risk counseling in subsequent pregnancies [1,3,4]. Within this agenda, the placenta is increasingly recognized as a key source of biologic evidence from pregnancy. It captures histopathologic signatures of impaired perfusion, inflammation, and abnormal villous development that may precede fetal demise [2-4].

Clinical guidance for stillbirth evaluation consistently identifies placental examination as a key component of the diagnostic workup because it can help explain the death, guide prevention planning, and inform surveillance in future pregnancies [5-7]. Standardized perinatal death classification systems also rely on attribution of placental and maternal fetal conditions to reduce unexplained stillbirth classification and improve comparability across settings [6]. However, the clinical interpretability of placental pathology depends on consistent lesion definitions, adequate sampling, and reproducible reporting across laboratories and clinical services [7].

A persistent challenge is that placental lesions are heterogeneous and have been inconsistently labeled across studies. This inconsistency limits cross-study synthesis and weakens inference about which findings are most informative for risk attribution. Consensus efforts, such as the Amsterdam Placental Workshop Group, have provided harmonized definitions and sampling recommendations. These support more reliable categorization of maternal vascular malperfusion, which reflects impaired uteroplacental perfusion and decidual vascular pathology; fetal vascular malperfusion, which reflects reduced perfusion of the villous tree and is often linked to cord compromise, stasis, or thrombosis; inflammatory lesions; and villous maturation abnormalities [8]. These malperfusion patterns plausibly converge on chronic hypoxia and terminal fetal decompensation [9,10].

Inflammation and immune-mediated placental disease represent additional pathways that may act independently or in combination with malperfusion. Contemporary pathologic frameworks describe acute chorioamnionitis and funisitis as staged maternal and fetal inflammatory responses that may occur with microbial invasion or sterile inflammation, with lesion timing and compartment involvement helping shape fetal risk [11]. Chronic inflammatory lesions, including villitis of unknown etiology, have also been implicated in placental dysfunction through villous injury and impaired exchange, although reported prevalence and effect estimates vary across populations and according to diagnostic criteria [12].

Recognition is increasing that placental disease contributes substantially to stillbirth. However, the evidence base remains fragmented. Studies differ in design, lesion taxonomy, comparator selection, and confounder adjustment strategies. This creates uncertainty about which histopathologic findings are most consistently linked to stillbirth and are most useful for clinical risk attribution [13,14]. Accordingly, the primary objective of this systematic review was to synthesize observational evidence on placental histopathologic lesions associated with stillbirth, with emphasis on recurring domain-level signals and comparative findings across standardized lesion categories. Rather than identifying a single dominant lesion pathway, the review aimed to clarify which placental pathologic domains were most consistently represented across the available evidence.

## Review

Materials and methods

Protocol and Reporting Standard

This systematic review was designed and reported in accordance with the PRISMA 2020 statement [15]. A protocol and analytic plan were prepared prior to screening and were not publicly deposited. No substantive deviations from the original analytic plan occurred during the review process.

Eligibility Criteria

The review question and eligibility criteria were structured using a prespecified Population, Intervention, Comparator, Outcome (PICO) framework that emphasized comparative studies evaluating placental histopathologic lesions in stillbirth versus live birth comparators, or versus other clearly defined nonstillbirth comparator groups. The PICO elements are summarized in Table 1.

**Table 1:** PICO framework and eligibility criteria Stillbirth definitions were accepted as reported by individual studies. Placental lesion categories were extracted as reported and synthesized across studies for narrative comparison. PICO: Population, Exposure/Index, Comparator, and Outcome

PICO element	Definition used in this review
Population (P)	Pregnant individuals with placentas examined after stillbirth, including antepartum or intrapartum fetal death, with a defined comparator group such as live births or other nonstillbirth outcomes.
Exposure/Index (I)	Placental histopathologic lesions reported by included studies, including lesion families such as malperfusion, infarction, thrombosis, inflammatory lesions, and villous maturation abnormalities.
Comparator (C)	Live birth placentas or other clearly defined nonstillbirth comparator groups.
Outcome (O)	Primary outcome: stillbirth or fetal death beyond the study-defined gestational age threshold. Secondary outcomes included lesion prevalence and crude or adjusted associations, such as odds ratios, risk ratios, prevalence ratios, or hazard ratios, when reported.
Eligible study designs	Analytical observational designs, including case control, cohort, and nested case control studies. Studies focused on placental pathology reporting methods were eligible only when they included stillbirth cases and contributed information relevant to lesion ascertainment or reporting heterogeneity.

We included analytical observational designs, including case-control, cohort, and nested case-control studies. Stillbirth definitions were accepted as reported in individual studies, typically using gestational age thresholds of 20-24 weeks, and each study's operational definition was extracted. Eligible studies were required to include stillbirth cases with placental examination and a defined comparator group, and to report placental histopathologic lesions with extractable lesion data or comparative results. Studies focused on placental pathology reporting methods were eligible only when they included stillbirth cases and contributed information relevant to lesion ascertainment or reporting heterogeneity. These studies were treated as contextual evidence and distinguished from those that provided primary evidence for lesion-outcome associations.

We excluded studies without a comparator group or studies that did not report placental histopathologic findings. We also excluded publication types without sufficient extractable data, such as narrative reviews, editorials, commentaries, protocols, and conference abstracts. Single case reports, noncomparative case series, animal studies, and reports where lesion data could not be linked to stillbirth outcomes were also excluded.

Information Sources and Search Strategy

We searched PubMed (MEDLINE), Embase, and Scopus from database inception through the final search update conducted in January 2025. The search combined controlled vocabulary, where available, and free text terms for stillbirth or fetal death, placenta or placental, histopathology or pathology, and lesion families including malperfusion, infarction, thrombosis, villitis, chorioamnionitis, and villous maturation abnormalities. The reference lists of included studies and relevant reviews were also examined to identify additional eligible records.

Full electronic search strategies were as follows:

PubMed (MEDLINE): (("Stillbirth"[Mesh] OR stillbirth*[tiab] OR "fetal death"[tiab] OR "intrauterine fetal death"[tiab] OR IUFD[tiab]) AND ("Placenta"[Mesh] OR placenta*[tiab] OR placental[tiab]) AND (histopatholog*[tiab] OR patholog*[tiab] OR "placental pathology"[tiab]) AND (malperfusion[tiab] OR "maternal vascular malperfusion"[tiab] OR "fetal vascular malperfusion"[tiab] OR infarct*[tiab] OR thrombo*[tiab] OR thrombosis[tiab] OR hemorrhag*[tiab] OR villitis[tiab] OR chorioamnionitis[tiab] OR funisitis[tiab] OR "villous maturation"[tiab] OR hypoxia[tiab] OR cord[tiab] OR membrane*[tiab]))

Embase: ('stillbirth'/exp OR stillbirth*:ti,ab OR 'fetal death':ti,ab OR 'intrauterine fetal death':ti,ab OR iufd:ti,ab) AND ('placenta'/exp OR placenta*:ti,ab OR placental:ti,ab) AND (histopatholog*:ti,ab OR patholog*:ti,ab OR 'placental pathology':ti,ab) AND (malperfusion:ti,ab OR 'maternal vascular malperfusion':ti,ab OR 'fetal vascular malperfusion':ti,ab OR infarct*:ti,ab OR thrombo*:ti,ab OR thrombosis:ti,ab OR hemorrhag*:ti,ab OR villitis:ti,ab OR chorioamnionitis:ti,ab OR funisitis:ti,ab OR 'villous maturation':ti,ab OR hypoxia:ti,ab OR cord:ti,ab OR membrane*:ti,ab)

Scopus: TITLE-ABS-KEY(stillbirth* OR "fetal death" OR "intrauterine fetal death" OR IUFD) AND TITLE-ABS-KEY(placenta* OR placental) AND TITLE-ABS-KEY(histopatholog* OR patholog* OR "placental pathology") AND TITLE-ABS-KEY(malperfusion OR "maternal vascular malperfusion" OR "fetal vascular malperfusion" OR infarct* OR thrombo* OR thrombosis OR hemorrhag* OR villitis OR chorioamnionitis OR funisitis OR "villous maturation" OR hypoxia OR cord OR membrane*)

Study Selection and Data Extraction

After deduplication, records were screened by title and abstract against the eligibility criteria, and potentially relevant reports were then assessed for full text. Screening and full-text eligibility assessment were performed independently by two reviewers, and disagreements were resolved through discussion and consensus, with adjudication by an additional reviewer when needed. Data extraction was performed independently by two reviewers using a standardized form, and discrepancies were resolved through discussion and consensus. Data were extracted using a standardized form that captured study design and setting, population characteristics, stillbirth definition, comparator definition, placental sampling and reporting approach, lesion definitions, and quantitative results, including lesion prevalence and effect estimates. When multiple effect measures were reported, adjusted estimates were prioritized. When adjustment sets differed across studies, covariates included in each model were extracted and considered during narrative interpretation of between-study differences to support interpretability and comparison across studies. Reasons for full-text exclusion were recorded systematically during the review process.

Risk of Bias Assessment

Risk of bias in included observational studies was assessed using the Newcastle-Ottawa Scale, with appropriate versions for case-control and cohort studies [16]. Disagreements in appraisal were resolved by consensus. Risk of bias was considered during the narrative synthesis, giving greater weight to findings that were consistent across studies with stronger internal validity.

Data Synthesis

Because clinical and methodological heterogeneity was anticipated across lesion taxonomies, comparator selection, gestational age composition, and effect measure reporting, synthesis was planned as a structured narrative supported by tabulation of results by lesion domain. Meta-analysis was considered only when lesion definitions, comparators, outcome definitions, and effect measures were sufficiently aligned to support meaningful pooling.

Ethics Statement

Ethics approval was not required because this study was a systematic review of published literature and did not involve direct contact with human participants or access to identifiable individual-level data.

Results

Study Selection

**Figure 1:**
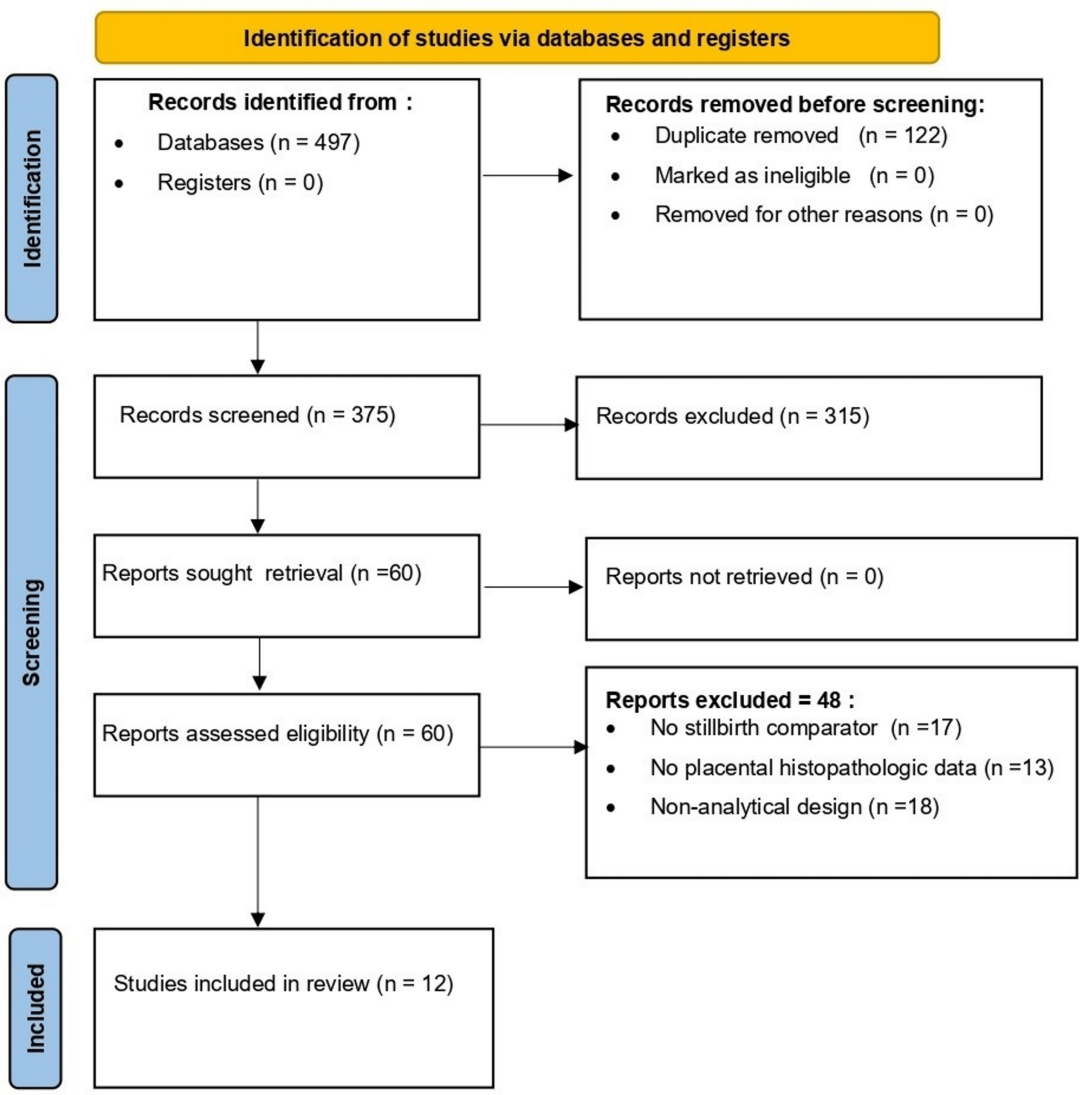
PRISMA 2020 flow diagram of study selection Flow diagram illustrating identification, screening, eligibility assessment, and inclusion of studies according to PRISMA 2020 guidelines. Numerical details of records identified, screened, excluded, and included are reported in the Results section.

The electronic search identified 497 records. After removing 122 duplicates, 375 unique records remained for title and abstract screening, of which 315 were excluded. Sixty full-text articles were assessed for eligibility; none were unretrievable. Forty-eight full-text articles were excluded because they lacked a stillbirth comparator (n=17), did not report placental histopathologic data (n=13), or used a non-analytical design (n=18). Ultimately, 12 studies were included in the qualitative synthesis (Figure 1).

Study Characteristics

The 12 included studies [17-28] comprised population-based and network case-control analyses, single-center and multicenter cohort studies, including one large prospective multicountry cohort. Some studies primarily estimated stillbirth associations, whereas others mainly informed lesion reporting methods and ascertainment rather than using a classical case-control contrast. Most analytic studies compared stillbirth placentas with live birth controls and evaluated lesions spanning vascular malperfusion, villous maturation, and hypoxia-pattern histology, inflammatory lesions, and cord or membrane abnormalities. Several studies applied structured lesion groupings aligned with contemporary placental pathology frameworks, whereas others used study-specific definitions or narrative reporting, contributing to heterogeneity in lesion ascertainment and reporting completeness. Study characteristics are summarized in Table 2.

**Table 2:** Characteristics of included studies (n=12)

Study	Design and setting	Comparator and sample	Main focus and lesion domains
Pinar et al., 2014 [17]	Network case-control analysis	Stillbirth versus live birth	Broad placental findings, including hemorrhage, thrombosis, infarction, inflammation, and cord abnormalities
Ptacek et al., 2016 [18]	Cohort with quantitative morphometry	Stillbirth and fetal growth restriction phenotypes versus comparison placentas	Morphometry, including syncytial nuclear aggregates, trophoblast area, and villous vascularity
Freedman et al., 2019 [19]	Case control	Stillbirth versus live birth	Adjusted associations for maternal and fetal circulatory disorder categories and inflammatory categories
Mecacci et al., 2016 [20]	Term case control	Term stillbirth versus low-risk term live births	Obstructive and disruptive lesions in term stillbirth
Jaiman et al., 2020 [21]	Cohort with controls	Fetal death versus control pregnancies	Villous maturation disorders, hypoxia pattern histology, maternal and fetal vascular malperfusion prevalence ratios
Patel et al., 2023 [22]	Hospital-based case-control	79 stillbirths versus 79 gestational age-matched live births	Odds ratios for vascular pathology, inflammation, calcification, and retroplacental clots
Ananthan et al., 2019 [23]	Case control	Stillbirth versus live birth controls	Lesion frequencies for fetal vascular malperfusion lesions, villous maturation lesions, villitis, and vasculitis
Kulkarni et al., 2021 [24]	Prospective multi-country cohort	Fetal death versus term live births	Maternal and fetal vascular malperfusion using consensus definitions with adjusted relative risks
Bukowski et al., 2017 [25]	Case-control and cohort analytic approach	Stillbirth versus live birth with newborn size strata	Placental findings in relation to stillbirth and newborn size strata
Dancey et al., 2023 [26]	Cross-sectional audit of reporting methods	Synoptic versus narrative reports	Effect of reporting format on lesion capture and agreement
Murvai et al., 2025 [27]	Cohort with phenotype clustering	Low placental growth factor pregnancies with adverse outcomes, including stillbirth	Placental phenotypes with maternal vascular malperfusion feature clustering in a high-risk population
Zur et al., 2024 [28]	Retrospective cohort with thrombophilia focus	Thrombophilia versus non-thrombophilia	Maternal vascular malperfusion type lesions and composite histologic scoring in a mechanistic subgroup

Main Findings

Across studies that directly compared stillbirth with live birth controls, placental abnormalities were frequently reported across vascular, inflammatory, villous maturation, and cord-related domains. Pinar et al. reported higher frequencies of abnormalities in stillbirth compared with live birth, including vascular degenerative changes, retroplacental hematoma, thrombi, infarction, acute chorioamnionitis, fetal vascular thrombi, and cord abnormalities, and they described differences in lesion patterns by gestational timing [17]. Ptacek et al. used quantitative histomorphometry and described a phenotype consistent with hypoxia and growth restriction, characterized by increased syncytial nuclear aggregates and trophoblast area with reduced villous vascularity, emphasizing that quantitative placental features may complement categorical lesion reporting [18]. In term stillbirths, Mecacci et al. reported enrichment of obstructive lesions, including infarcts and thrombi, compared with low-risk term controls, supporting an obstructive and hemorrhagic signal in later gestation stillbirth [20].

Using lesion category groupings in adjusted analyses, Freedman et al. reported elevated odds of stillbirth associated with maternal circulatory disorder categories and fetal circulatory disorder categories, and they also reported an association with maternal inflammatory response categories [19]. Jaiman et al. reported markedly higher prevalence of villous maturation disorders in fetal death compared with controls and also reported higher prevalence of hypoxia pattern histology and fetal vascular malperfusion lesions, with a smaller contrast reported for chronic inflammatory lesions [21]. Patel et al. reported high odds ratios for retroplacental clots, uteroplacental vascular pathology, calcific changes, acute chorioamnionitis, and chronic inflammation in a hospital-based case-control study with gestational age-matched controls [22].

Ananthan et al. reported higher frequencies of fetal vascular malperfusion lesions, delayed villous maturation, and inflammatory lesions, including villitis and chorionic plate vasculitis, in stillbirth compared with live birth controls [23]. In a prospective multicountry cohort using consensus lesion definitions, Kulkarni et al. reported increased frequency of both maternal vascular malperfusion and fetal vascular malperfusion in fetal deaths compared with term live births, with elevated relative risks reported for both domains [24]. Bukowski et al. reported associations between stillbirth and multiple placental findings and demonstrated that interpretation differed across newborn size strata, highlighting the importance of fetal growth context when assessing lesion and outcome relationships [25].

Two included studies primarily informed the interpretation of between-study heterogeneity rather than providing direct stillbirth versus live birth effect estimates for individual lesions. Dancey et al. reported that synoptic reporting improved lesion capture and agreement compared with narrative reporting, highlighting the reporting format itself as a contributor to variation in lesion prevalence across studies [26]. Zur et al. described placental phenotypes in pregnancies with low placental growth factor and linked maternal vascular malperfusion feature clustering with adverse outcomes, including stillbirth, in a high-risk population, but the study's focus was phenotype structure rather than stillbirth-specific lesion effect estimation [28]. Murvai et al. examined maternal vascular malperfusion type lesions and composite histologic scoring in relation to hereditary thrombophilia, informing mechanistic subgrouping rather than providing a stillbirth comparator effect measure [27].

Because lesion definitions, comparators, gestational age composition, and effect measures varied across studies, meta-analysis was not performed. Instead, extracted comparative association measures were summarized for studies that reported explicit odds ratios, risk ratios, or prevalence ratios (Table 3).

**Table 3:** Extracted comparative association estimates for stillbirth versus comparator groups, where reported CI: confidence interval; OR: odds ratio; PR: prevalence ratio; RR: risk ratio

Study	Domain or lesion measure	Effect estimate as reported
Freedman et al., 2019 [19]	Maternal circulatory disorder category	Adjusted OR 4.14, 95% CI 2.93 to 5.84
Freedman et al., 2019 [19]	Fetal circulatory disorder category	OR 4.58, 95% CI 3.11 to 6.74
Freedman et al., 2019 [19]	Maternal inflammatory response category	Adjusted OR 2.58, confidence interval not available in the extracted data
Kulkarni et al., 2021 [24]	Maternal vascular malperfusion	Adjusted RR 3.88, 95% CI 2.70 to 5.59
Kulkarni et al., 2021 [24]	Fetal vascular malperfusion	RR 4.09, 95% CI 2.15 to 7.75
Jaiman et al., 2020 [21]	Villous maturation disorders	PR 44.6, confidence interval not available in the extracted data
Jaiman et al., 2020 [21]	Hypoxia pattern histology	PR 6.8, confidence interval not available in the extracted data
Jaiman et al., 2020 [21]	Fetal vascular malperfusion	PR 4.5, confidence interval not available in the extracted data
Jaiman et al., 2020 [21]	Chronic inflammatory lesions	PR 1.8, confidence interval not available in the extracted data
Patel et al., 2023 [22]	Retroplacental clots	OR 9.95, 95% CI 4.39 to 11.71
Patel et al., 2023 [22]	Uteroplacental vascular pathology	OR 7.39, 95% CI 3.01 to 8.97
Patel et al., 2023 [22]	Calcific changes	OR 4.46, 95% CI 2.56 to 6.01
Patel et al., 2023 [22]	Acute chorioamnionitis	OR 3.35, 95% CI 2.11 to 5.21
Patel et al., 2023 [22]	Chronic inflammation	OR 2.33, 95% CI 1.91 to 4.17

Risk of Bias Within Included Studies

Using the Newcastle-Ottawa Scale domains, internal validity for stillbirth association inference was strongest in larger population-based and prospective designs with clearer comparator definitions and standardized lesion ascertainment, and it was weaker in smaller single-center retrospective studies in which selection mechanisms, confounder control, and reporting completeness could plausibly influence observed lesion frequency and effect estimates. Accordingly, findings from studies with stronger internal validity were given greater interpretive weight in the narrative synthesis, whereas findings from smaller retrospective studies were interpreted more cautiously. The reporting methods audit was appraised for audit validity and was not interpreted as providing lesion and stillbirth causal inference (Table 4).

**Table 4:** Risk of bias summary using the Newcastle-Ottawa Scale domains at the study level judgment Overall judgment reflects internal validity for each study’s primary aim. For Dancey et al., the judgment reflects reporting audit validity rather than lesion and stillbirth association inference.

Study	Selection domain	Comparability domain	Exposure or outcome domain	Overall judgment for the study's primary aim
Pinar et al., 2014 [17]	Low to moderate	Moderate	Low to moderate	Moderate
Ptacek et al., 2016 [18]	Moderate	Moderate	Moderate	Moderate
Freedman et al., 2019 [19]	Moderate	Lower	Low to moderate	Moderate
Mecacci et al., 2016 [20]	Moderate	Moderate	Moderate	Moderate
Jaiman et al., 2020 [21]	Moderate	Moderate	Moderate	Moderate
Patel et al., 2023 [22]	Moderate to high	Moderate	Moderate	Moderate to high
Ananthan et al., 2019 [23]	Moderate	Moderate	Moderate	Moderate
Kulkarni et al., 2021 [24]	Lower	Lower	Moderate	Moderate
Bukowski et al., 2017 [25]	Moderate	Moderate	Moderate	Moderate
Dancey et al., 2023 [26]	Moderate	Not applicable	Moderate	Moderate, audit validity
Murvai et al., 2024 [27]	Moderate to high	Moderate	Moderate	Moderate to high
Zur et al., 2025 [28]	Moderate	Moderate	Moderate	Moderate

Discussion

Principal Findings

This systematic review found that stillbirth is associated with placental pathology across multiple domains rather than a single dominant lesion pattern. Across included studies, vascular malperfusion, obstructive or hemorrhagic lesions, villous maturation and hypoxia pattern abnormalities, inflammatory lesions, and cord related abnormalities were consistently reported more frequently in stillbirth than in comparator pregnancies, although the magnitude and specificity of these associations varied according to study design, lesion taxonomy, comparator selection, and the gestational age composition of the study populations [17-25]. Population-based and network studies documented large absolute contrasts for several clinically recognizable lesions, whereas studies using quantitative morphometry or phenotype clustering suggested that chronic placental compromise may also manifest as measurable structural remodeling rather than only as discrete categorical lesions [17,18,27]. Overall, the evidence supports a convergent model in which impaired uteroplacental and fetoplacental perfusion, abnormal villous development, and inflammation contribute to stillbirth risk, often with lesion co-occurrence rather than isolated mechanistic pathways.

Vascular Malperfusion and Obstructive or Hemorrhagic Injury

A consistent signal across studies is enrichment of vascular malperfusion and obstructive or hemorrhagic injury patterns in stillbirth. Term-focused evidence highlighted obstructive lesions, with Mecacci et al. reporting infarcts and thrombi in 54.6% of term stillbirth placentas compared with 7.5% of low-risk term live births [20]. In a hospital-based case-control study with gestational age-matched controls, Patel et al. reported high odds ratios for retroplacental clots and uteroplacental vascular pathology, alongside other lesions, indicating that vascular and hemorrhagic findings can be prominent in routine clinical datasets [22]. In a prospective multicountry cohort using consensus definitions, Kulkarni et al. reported elevated relative risks for both maternal vascular malperfusion and fetal vascular malperfusion in fetal death compared with term live birth controls, supporting the reproducibility of malperfusion associations across settings when taxonomy is standardized [24]. Differences in analytic framing may explain variation in how vascular pathology is presented. Freedman et al. reported elevated odds for maternal circulatory and fetal circulatory disorder categories and an association for maternal inflammatory response categories, reflecting a domain-based approach that can retain independent maternal and fetal circulatory contributions after adjustment rather than attributing risk to single lesions [19].

Overall, these findings support vascular malperfusion as a central pathway in stillbirth, while also indicating that the apparent dominance of specific vascular lesions depends on gestational age restriction, comparator baseline risk, and whether lesions are grouped into domains or evaluated individually.

Villous Maturation Abnormalities, Hypoxia Pattern Histology, and Quantitative Remodeling

Villous maturation abnormalities and hypoxia pattern histology produced some of the largest reported contrasts in studies that applied explicit maturation frameworks. Jaiman et al. reported villous maturation disorders in 44.0% of fetal deaths compared with 1.0% of controls, with a prevalence ratio of 44.6, and they also reported hypoxia pattern histology and fetal vascular malperfusion as more prevalent among fetal deaths [21]. These estimates highlight villous developmental and chronic hypoxic adaptation pathways as potentially high signal features when they are consistently defined and actively sought. Ptacek et al. extended this concept by demonstrating that stillbirth-associated placental compromise may also be reflected in quantitative morphometry, including greater syncytial nuclear aggregates and trophoblast area with reduced villous vascularity in a growth-restricted stillbirth phenotype, suggesting that continuous measures may complement categorical lesion classification in selected phenotypes [18]. Variation across studies in villous maturation estimates is likely driven by differences in diagnostic criteria, sampling intensity, and the baseline risk profile of comparator populations. This emphasizes the importance of reporting explicit definitions and control characteristics when interpreting villous maturation findings across studies.

Inflammation and Its Co-occurrence With Vascular Lesions

Inflammatory lesions were reported across several datasets, but their interpretation requires attention to both the co-occurrence with vascular pathology and differences in ascertainment. Pinar et al. reported higher frequencies of acute chorioamnionitis of membranes in stillbirth compared with live birth, alongside multiple vascular, thrombotic, and hemorrhagic abnormalities, indicating that inflammation commonly appears within a broader lesion context rather than as an isolated finding [17]. Patel et al. reported increased odds for acute chorioamnionitis and chronic inflammation in placentas, again occurring alongside strong associations for vascular and hemorrhagic pathology [22]. In a lesion-rich case-control comparison, Ananthan et al. reported higher frequencies of inflammatory and immune-mediated lesions in stillbirth than live birth controls, together with fetal vascular malperfusion lesions and delayed villous maturation, thereby illustrating lesion clustering across domains [23]. By contrast, the prospective multicountry cohort by Kulkarni et al. emphasized standardized vascular malperfusion domains as stable predictors, which may reflect both the taxonomic focus of the study and the greater stability of malperfusion ascertainment under consensus definitions [24]. These observations suggest that inflammation may represent a primary causal pathway in some cases and a secondary or co-traveling marker in others. Future comparative studies are likely to be more informative when they jointly model inflammation, malperfusion, gestational age, and fetal growth rather than evaluating each domain in isolation.

Heterogeneity, Comparator Selection, and Reporting Completeness

A major source of uncertainty in the evidence base is methodological heterogeneity. Studies differed in lesion taxonomy, sampling protocols, comparator selection, and confounder control, all of which can meaningfully change lesion prevalence and association estimates. Bukowski et al. demonstrated that interpretation of placental findings can differ across newborn size strata, reinforcing that fetal growth context can modify apparent lesion associations and should be incorporated into analytic models when possible [25]. Dancey et al. showed that synoptic reporting improved lesion capture compared with narrative reporting, supporting reporting format as a plausible contributor to between-study variability in lesion prevalence and potentially to misclassification of exposure status in retrospective datasets [26]. Together, these findings indicate that a portion of apparent biological heterogeneity likely reflects differences in measurement and documentation rather than true differences in pathophysiology.

High-Risk Phenotyping and Mechanistic Subgrouping

Two included studies contributed primarily to mechanistic interpretation rather than direct estimation of lesion-specific stillbirth effects. Zur et al. identified distinct placental phenotypes in pregnancies with low placental growth factor and linked maternal vascular malperfusion feature clustering with adverse outcomes, including stillbirth, within a biomarker-defined high-risk population, supporting phenotype-oriented approaches in selected clinical contexts [28]. Murvai et al. associated maternal vascular malperfusion type lesions and a composite histologic score with hereditary thrombophilia, suggesting that placental vascular injury patterns may reflect maternal prothrombotic biology in some subgroups, although an effect estimate for stillbirth association was not extracted from that cohort [27]. These studies emphasize that similar downstream placental injury patterns may arise from different upstream maternal drivers, supporting integrated models that incorporate maternal clinical context alongside standardized placental lesion reporting.

Limitations

This review has limitations that affect the strength and generalizability of inferences. First, substantial clinical and methodological heterogeneity across studies reduced direct comparability and precluded meta-analysis. Second, stillbirth definitions, gestational age composition, sampling protocols, and lesion taxonomy varied, increasing the risk of exposure misclassification and limiting harmonization. Third, confounder adjustment strategies were inconsistent, and residual confounding likely remained in several observational designs, particularly for maternal comorbidities, fetal growth restriction, and gestational age. Fourth, not all studies reported confidence intervals for all extracted measures, limiting precision assessment for some associations. Fifth, the final search update was conducted in January 2025, which may omit more recent studies. Sixth, the protocol was not publicly registered, which can increase the perceived risk of selective reporting even when an a priori plan existed. Finally, publication bias and selective outcome reporting cannot be excluded.

## Conclusions

This systematic review synthesizes observational evidence indicating that stillbirth is associated with placental pathology across multiple domains rather than a single dominant lesion pattern. Vascular malperfusion and obstructive or hemorrhagic lesions were the most consistently reported domains, with additional recurrent signals involving villous maturation and hypoxia pattern abnormalities, as well as inflammatory lesions that frequently co-occurred with vascular findings.

Interpretation across studies is limited by heterogeneity in lesion taxonomy, sampling intensity, comparator selection, and confounder adjustment, which constrained quantitative pooling. Future work using standardized lesion definitions, reproducible sampling and synoptic reporting, and analyses stratified by gestational age and fetal growth context is likely to improve comparability, strengthen inference regarding the most informative placental findings for stillbirth attribution, and enhance recurrence risk counseling.
